# Chronic Liver Disease in Ethiopia with a Particular Focus on the Etiological Spectrums: A Systematic Review and Meta-Analysis of Observational Studies

**DOI:** 10.1155/2021/8740157

**Published:** 2021-11-23

**Authors:** Behailu Terefe Tesfaye, Temesgen Mulugeta Feyissa, Azmeraw Bekele Workneh, Esayas Kebede Gudina, Mengist Awoke Yizengaw

**Affiliations:** ^1^Jimma University, Institute of Health, School of Pharmacy, Clinical Pharmacy Unit, Jimma, Ethiopia; ^2^Jimma University Medical Center, Institute of Health, Jimma, Ethiopia; ^3^Jimma University, Institute of Health, School of Pharmacy, Social Pharmacy Unit, Jimma, Ethiopia; ^4^Jimma University, Institute of Health, Department of Internal Medicine, Jimma, Ethiopia

## Abstract

**Background:**

In Ethiopia, chronic liver disease (CLD) is the 7th leading cause of death, accounting for about 24 deaths per 100000 populations in 2019. Despite its burden, there is a lack of compiled pieces of evidence on CLD in the country. Thus, this systematic review and meta-analysis is intended to provide the pooled estimates of CLD etiologies and mortality rate in CLD patients in Ethiopia.

**Method:**

PubMed, Google Scholar, ScienceDirect, institutional repositories, national digital library, and the bibliography of the eligible articles information were the source of data for the present review. The keywords “hepatitis, chronic” [Mesh], “end-Stage Liver Disease” [Mesh], “chronic liver disease”, “liver cirrhosis” [Mesh], and “Ethiopia” were used for the searches. Overall, we retrieved 199 records and 12 were included in this review. We used the DerSimonian-Laird random-effects models to perform the meta-analysis. We conducted subgroup and meta-regression analyses to account for the heterogeneity of the estimates.

**Result:**

Hepatitis B virus, alcohol, and hepatitis C virus are the three most common etiologies of CLD in Ethiopia accounting for a pooled estimate of 40.0% [95% CI: 29.0, 51.0, *I*^2^ = 96.3, *p* < 0.001], 17.0% [95% CI: 9.0, 25.0, *I*^2^ = 96.7, *p* < 0.001], and 15.0% [95% CI: 9.0, 21.0, *I*^2^ = 95.8, *p* < 0.001], respectively. Unidentified etiology report has a substantial contribution accounting for an estimated pooled proportion of 45% [95% CI: 34.0, 56.0%, *Q* = 32.08, *p* < 0.001, *I*^2^ = 87.53] of the CLD cases in the country. On the other hand, the overall hospital mortality rate in CLD patients is 25.0% [95% CI: 2.0, 47.0, I^2^ = 94.6, *p* < 0.001] in Ethiopia.

**Conclusion:**

Hepatitis B virus, hepatitis C virus, and alcohol are the three most common contributors to CLD cases in Ethiopia. The authors warrant routine screening and strengthening of preventive and treatment programs for viral hepatitis B and C, further enhancing the alcohol policy of the country.

## 1. Introduction

Chronic liver disease (CLD) is a progressive decline in liver function, including the synthesis of important proteins, detoxification of metabolites, and excretion of bile, lasting for over 6 months [1, 2]. It involves a range of pathologies, such as chronic hepatitis, cirrhosis, and hepatocellular carcinoma [2, 3]. CLD is a global public health problem affecting about 1.5 billion individuals worldwide in 2020 [4]. Cirrhosis, which is fibrosis of the liver due to long-term liver damage [5], has contributed to 2.4% of total deaths globally in 2017 [6]. This disease impairs the health-related quality of life of the affected individuals causing different clinical manifestations, including pruritus, joint pain, abdominal pain, muscle cramps, fatigue, depression, and anxiety [7, 8].

There are various etiologies of CLD. Chronic alcohol intake, viral hepatitis B, C, and D infections, nonalcoholic fatty liver disease (NAFLD), autoimmune hepatitis, and drugs are the common ones identified across the world [9]. Globally, the proportion of CLD caused by hepatitis B virus (HBV) is 3.6%, ranging from 0.5% in European countries to over 8% in sub-Saharan Africa, while hepatitis C virus (HCV) contributed 2.5% of the CLD causes ranging from 1.8% in the United States (US) to 5.6% in Africa. The magnitude of alcohol-caused CLD is 8.5% with the highest report (around 12%) being from Europe and the United States. Similarly, NAFLD accounted for 25% of the CLD causes globally, with the highest report from western countries (17% to 46%) [10]. In Africa, a study from Togo has reported HBV (66%), alcohol abuse (57%), and HCV (12.3%) as the most common etiologies of CLD [11].

Even though advances have been made in the diagnosis and treatment of liver disease, the burden of CLD is increasing worldwide [12, 13]. Cirrhosis is the 7th leading cause of mortality in Ethiopia, accounting for 24 deaths per 100000 population in 2019 [14]. This shows the necessity of implementing cost-effective prevention and treatment programs in the country. For the effectiveness of the prevention and treatment programs, investigating the etiological distribution of CLD plays a pivotal role [15]. Despite this, there is a lack of comprehensive epidemiological data on the etiological distribution of CLD in Ethiopia. Therefore, this systematic review and meta-analysis aimed to provide a combined estimate of CLD etiologies and mortality rate in CLD patients in Ethiopia.

## 2. Method

This is a systematic review and meta-analysis of articles based on observational studies reporting on CLD etiologies from Ethiopia. A Preferred Reporting Items for Systematic Reviews and Meta-Analyses (PRISMA) flow checklist 2020 [16] was strictly followed in designing and reporting. The protocol of this review is registered [CRD42021264454] on PROSPERO [https://www.crd.york.ac.uk/prospero/display_record.php?ID=CRD42021264454].

### 2.1. Eligibility Criteria

#### 2.1.1. Inclusion and Exclusion Criteria

Both journal articles and thesis works involving humans, conducted in Ethiopia and reported in English, were included in this review. We excluded articles lacking sufficient information (with no full-text), review articles, and publications from personal opinions/conference presentations.

### 2.2. Data Sources

PubMed [17], ScienceDirect [18], Google Scholar [19], institutional repositories of Addis Ababa [20] and Jimma University [21], the national academic digital library [22], and bibliographies of relevant articles were the sources of data for this review.

### 2.3. Search Strategy

We searched major databases (PubMed, Google Scholar, and ScienceDirect), institutional repositories, national digital libraries, and bibliographies of relevant articles. We conducted the searches from June 06 to 10, 2021. The keywords employed during the searches were “Hepatitis, Chronic” [Mesh], “End-Stage Liver Disease” [Mesh], “chronic liver disease”, “Liver Cirrhosis” [Mesh], and “Ethiopia”. In searching from the databases, we linked the keywords using Boolean operators (“AND,” “OR”). The search detail for PubMed was ((((“Hepatitis, Chronic” [Mesh]) OR (“End-Stage Liver Disease” [Mesh])) OR (chronic liver disease)) OR (“Liver Cirrhosis”[Mesh])) AND (“Ethiopia” [Mesh]) restricting to human studies and articles reported in English. We used a similar search detail in searching from ScienceDirect too. The search detail for Google scholar was all in the title: “Hepatitis, Chronic” OR “End-Stage Liver Disease” OR “chronic liver disease” OR “Liver Cirrhosis” OR “Chronic hepatitis” “Ethiopia”. We explored the national and institutional repositories, searching each keyword at a time. A manual search of bibliography lists from all the eligible articles was also carried out to identify further potentially eligible articles.

### 2.4. Study Selection Process

We imported results of the search from the databases and stored them in the Mendeley reference manager, and duplicates were recorded and removed. Three authors (BT, MA, and AB) performed screening of the retrieved records based on title and abstract. While screening, they categorized the records as either “yes”, “maybe”, or “no”. Full-text of studies considered as “yes” or “maybe” during the screening were further assessed based on the eligibility criteria by two authors (TM and BT). In each case, the third author (EK) played a role in solving discrepancies that arose between the two authors.

### 2.5. Data Extraction

We extracted data using a standard format adapted from Joanna Briggs Institute (JBI). The format was prepared on Microsoft Excel. Three authors (BT, MA, and TM) independently extracted relevant data from each eligible study (author name, publication/study year, study setting, study design, study population, sample size, age, and sex) and reported CLD etiologies and mortality rate. They exchanged the extracted data for cross-checking. In case of any disagreement, EK consulted for a resolution.

### 2.6. Quality Assessment of the Studies

Two authors (TM and AB) independently assessed the quality of each study, and they resolved disagreements arising during the assessment through discussion with BT. We assessed all the included studies for methodological quality using the JBI critical appraisal checklist tool [23]. The evaluation content includes the sample inclusion criteria, the study subjects and the description of the setting(s), exposure measurement, the objective and standard criteria used for measurement of the condition, confounding factors identification, the strategies to deal with confounding factors, measurement of the outcomes, and the statistical analysis used. Each component was rated as “yes” or “no”. Most of the studies [24–28] have problems related to the identification of the confounding variables and the lack of strategies for dealing with the confounders. Similarly, an appropriate statistical analysis based on the study objectives was not performed in some studies [24, 25, 27–30]. In the present systematic review and meta-analysis, we included all articles fulfilling at least an average score (≥50%) of “yes” on the JBI quality assessment tool (Table 1).

### 2.7. Data Analysis and Synthesis

In this review, the term etiology implies causes or risk factors that are associated with an increased likelihood of CLD occurrence, as reported in the primary studies. The estimated proportion of CLD etiology was the primary outcome, while the secondary outcome was mortality. After completing the data extraction process in excel, we exported the data to STATA 16.0 software for analysis. A Forest plot was used to depict the estimated proportion of most common CLD etiologies (HBV, HCV, and alcoholic CLD) and mortality with 95% CI. A heterogeneity (*I*^2^) test was conducted to examine the existence of dispersion among the proportion of the estimates with consideration of *I*^2^ values of 0, 25, 50, and 75% as no, low, moderate, and high (considerable) heterogeneity, respectively [36]. The DerSimonian-Laird random-effects model was selected for the analysis. In a meta-analysis, meta-regression should generally be considered when at least ten studies have reported the variable of interest [37]. Thus, in the present review, the authors performed meta-regression analysis only for the HBV and HCV estimates to identify the source of heterogeneity using age and sample size as covariates. We presumed there could be a variation in the distribution of CLD etiologies across the different study regions (northern, Addis Ababa, and Oromia) and after the implementation of the national strategy for the prevention and control of viral hepatitis in Ethiopia in 2016 [38]. Thus, we conducted a subgroup analysis based on the study regions (for HBV, HCV, and alcohol) and study year (before 2016 vs. since 2016). All the included studies reporting alcoholic etiology were conducted since 2016; thus, we did the subgroup analysis based on the study year only for HBV and HCV. We evaluated the possibility of publication bias through visual inspection of the funnel plot (for HBV, HCV, and alcoholic CLD depicted in the manuscript) and Egger's regression test. In all the analysis, two-sided *p* value < 0.05 was considered for declaring statistical significance.

## 3. Results

### 3.1. Study Selection

**Figure 1:**
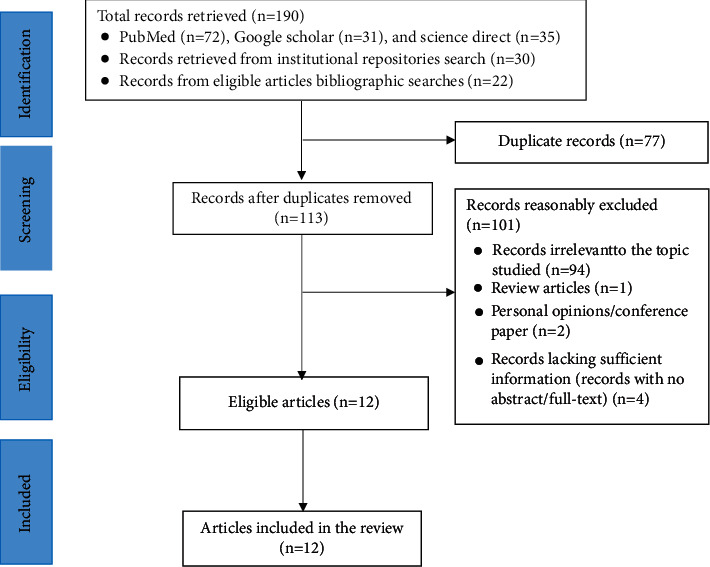
PRISMA flow chart showing studies retrieved, screened, and included.

The searches from major databases, institutional repositories, and eligible articles' bibliography resulted in 190 records. After removing duplicates and reasonable exclusion of records, 12 were included in the review [24–35]. Of the twelve, two [32, 33] studies represent the same population data and one with additional CLD etiology reports; thus, the additional etiology reports were extracted and analyzed, leaving the duplicate reports from the second article (Figure 1).

### 3.2. Study Characteristics

In this review, we included nine journal articles [24–26, 28, 29, 31–34] and three thesis works [27, 30, 35]. All the included studies are observational: eight cross-sectional [24–28, 30, 32, 34] and four case-control [29, 31, 33, 35]. Except for one [29], all were conducted since 2014. In terms of the study regions, most of them were from Addis Ababa [25, 26] (Table 2).

### 3.3. Publication Bias Assessment

**Figure 2:**
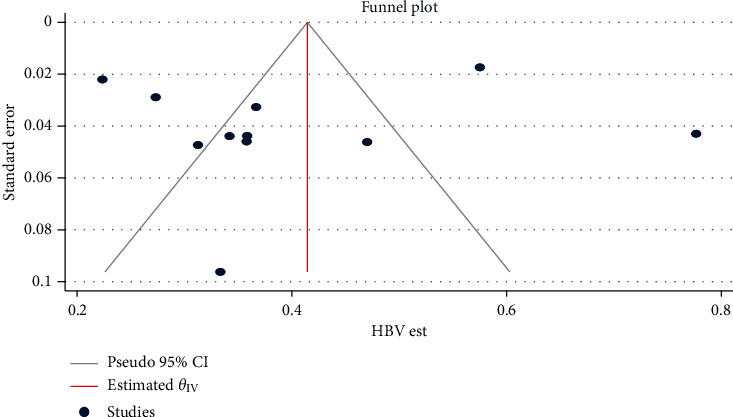
Funnel plot for assessing publication bias across HBV estimates among chronic liver disease patients in Ethiopia, 2021.

**Figure 3:**
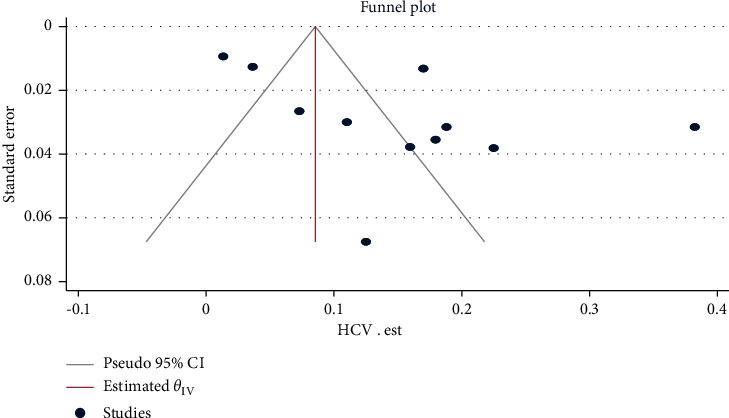
Funnel plot for assessing publication bias across HCV infection estimates among chronic liver disease patients in Ethiopia, 2021.

The funnel plots of the estimates are symmetry showing the absence of publication bias across the studies, except for alcoholic, co-hepatitis B and C infection, and mortality estimates. Egger's regression test also statistically confirmed these findings: HBV (*β* = −0.51, 95% CI: −6.51, 5.48, *p*=0.866) (Figure 2), HCV (*β* = 2.61, 95% CI: −1.03, 6.25, *p*=0.062) (Figure 3), hepatosplenic schistosomiasis (*β* = 1.26, 95% CI: -1.52, 4.05, *p*=0.375), and unidentified etiology (*β* = −0.30, 95% CI: −6.39, 5.79, *p*=0.923).

**Figure 4:**
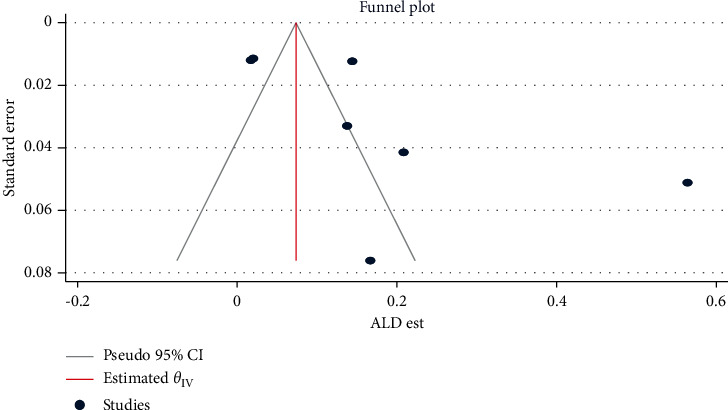
Funnel plot for assessing publication bias across alcoholic hepatitis estimates among chronic liver disease patients in Ethiopia, 2021.

**Figure 5:**
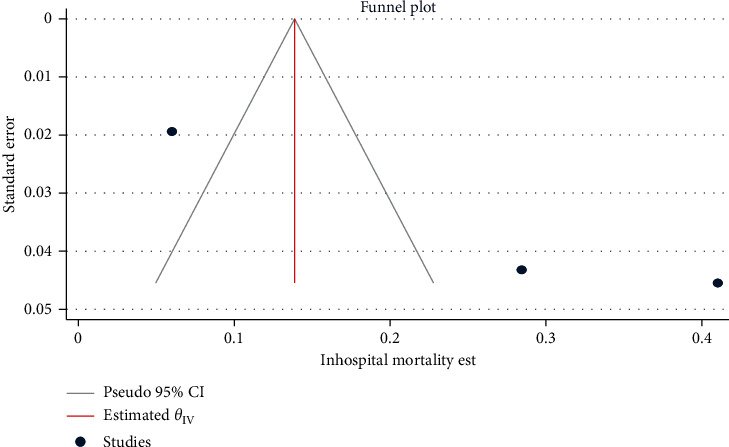
Funnel plot for assessing publication bias across mortality estimates among chronic liver disease patients in Ethiopia, 2021.

Alcoholic hepatitis (*β* = 4.75, 95% CI: 1.56, 7.94, *p*=0.003) (Figure 4), co-hepatitis B and C infection (*β* = 1.83, 95% CI: 0.175, 3.477, *p*=0.032), and mortality (*β* = 11.68, 95% CI: 5.68, 17.68, *p* < 0.001) (Figure 5) estimates had a statistically significant publication bias.

### 3.4. Results of Individual Studies

Among the reported etiologies of CLD, hepatitis B and C virus were noted in all the included studies [24–35], while alcohol was reported in eight studies [24, 25, 27, 31–35] The highest report of HBV (78.0%) was from Mekelle [35], while a study from Bale Robe [28] reported the lowest proportion (22.0%). For HCV, a study from Addis Ababa [29] recorded the highest contribution (38.0%), while the lowest (1.0%) report was from Harar [32]. From the noninfectious etiologies of CLD, the report of alcoholic etiology was the highest (56.0%) in a study from Mekelle [35], while reports of studies from Addis Ababa [25] and Harar [32] were the lowest (2.0%). Nonalcoholic fatty liver disease was reported in two studies [31, 34] with the higher (20.0%) report from Addis Ababa [31] and the lower (4.0%) from Jimma [34]. In multiple studies, we captured reports of unidentified etiology ranging from 26.0% in a study from Jimma [34] to 55.0% from Harar [32]. One study from Harar [33] identified khat chewing as the only risk factor in 82.5% of the participants with unidentified etiology. Some studies [25–27, 29, 30, 34] had reported concurrent CLD etiologies, of which, co-hepatitis B and D virus [29] showed the highest rate, 23.1% (15/65), as compared to other concurrent CLD etiologies reports (Table 3).

### 3.5. Pooled Estimated Proportion and Heterogeneity

**Figure 6:**
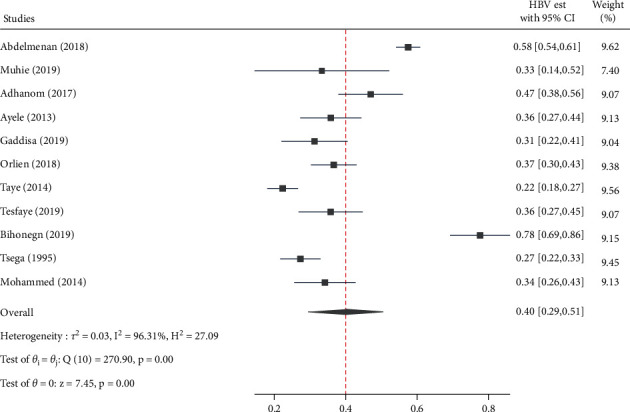
Forest plot depicting the estimated proportion of HBV infection among chronic liver disease patients in Ethiopia, 2021.

**Figure 7:**
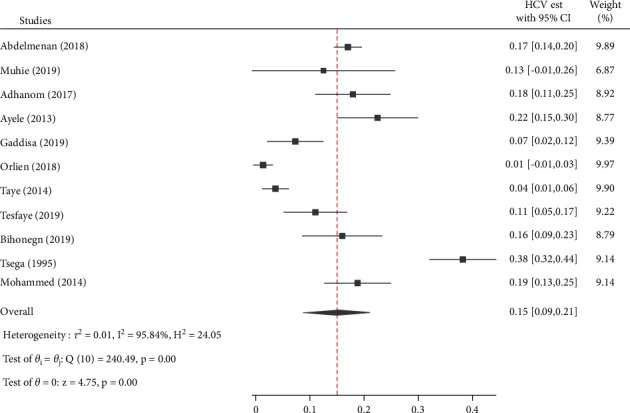
Forest plot depicting the estimated proportion of HCV infection among chronic liver disease patients in Ethiopia, 2021.

The overall pooled estimated proportion was 40.0% [95% CI: 29.0, 51.0, *Q* = 270.9, *p* < 0.001, *I*^2^ = 96.3] for HBV (Figure 6), while HCV has contributed 15.0% [95% CI: 9.0, 21.0, *Q* = 240.4, *p* < 0.001, *I*^2^ = 95.8] (Figure 7) to CLD cases in Ethiopia.

**Figure 8:**
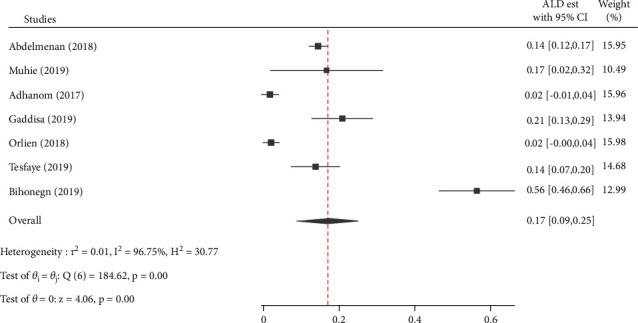
Forest plot depicting the estimated proportion of alcoholic hepatitis among chronic liver disease patients in Ethiopia, 2021.

Alcohol was the third most commonly reported CLD etiology with the second-highest estimated pooled proportion, 17.0% [95% CI: 9.0, 25.0, *Q* = 184.6, *p* < 0.001, *I*^2^ = 96.7] (Figure 8).

Besides, NAFLD has a 12.0% [95% CI: −4.0, 28.0, *Q* = 51.6, *I*^2^ = 98.0, *p* < 0.001] to CLD cases in the country. Unfortunately, unidentified etiology report has contributed substantially accounting for an estimated pooled proportion of 45% [95% CI: 34.0, 56.0%, *Q* = 32.08, *p* < 0.001, *I*^2^ = 87.53] of CLD cases in the country. On the other hand, the present study revealed a smaller contribution of hepatosplenic schistosomiasis, Wilson's disease, autoimmune hepatitis (AIH), and human immunodeficiency virus (HIV) to CLD cases in the country. From the concurrent etiologies of CLD, only co-hepatitis B and C virus infection was reported in multiple studies accounting for an estimated rate of 1.0% [95% CI: 0.0, 2.0, *Q* = 3.7, *p*=0.30, *I*^2^ = 18.9] (Table 4).

On multivariate meta-regression, we identified a statistically significant correlation of HCV estimates with the age of the participants [AOR = 0.016, 95% CI: 0.001, 0.031, *p*=0.03] implying less likelihood of HCV with an increase in age (Table 5).

### 3.6. Subgroup Analysis of Chronic Liver Disease Etiology Estimates

The estimated pooled proportion of HCV is significantly the highest in studies from Addis Ababa, 23.0% [95% CI: 15, 30.0, *p* < 0.001], while the estimated pooled proportion of HBV was significantly higher in those studies conducted since 2016 as compared to those before 2016 (46.0% vs. 29.0%, *p*=0.01), whereas the estimate is higher in those conducted before 2016 for HCV (27.0% vs. 12.0%, *p*=0.33) (Table 6).

### 3.7. Reported Mortalities in Chronic Liver Disease Patients

**Figure 9:**
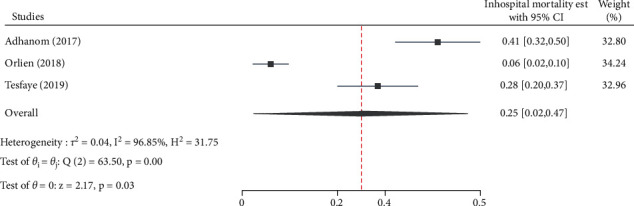
Forest plot depicting the estimated proportion of mortality among chronic liver disease patients in Ethiopia, 2021.

The overall pooled estimate of inpatient mortality in CLD patients is 25.0% [95% CI: 2.0%, 47.0%, *p* < 0.001, *Q* = 63.5, *p* < 0.001, *I*^2^ = 96.8] in Ethiopia (Figure 9).

## 4. Discussion

This systematic review and meta-analysis is the first in Ethiopia to compile the etiological spectrums of CLDs and mortality rates of CLD patients in the country.

Chronic liver disease is a known public health problem causing substantial morbidity and mortality across the globe [39]. There are different etiologies of CLD, and the relative contribution of these etiologies follows a geographical pattern [40]. The three most common etiologic contributors of CLD identified in the present review were HBV, alcohol, and HCV accounting for 40.0% (range: 22.0% to 78.0%), 17.0% (range: 2.0% to 56%), and 15.0% (range: 1.0% to 38.0%), respectively. Consistent with our finding, a study from Togo has reported HBV (66%), alcohol abuse (57%), and HCV (12.3%) as the three principal causes of CLD [11]. Globally, HBV and HCV are the major cause of CLD [6, 41]. Similarly, viral hepatitis B and C are the predominant etiologies of CLD in eastern European countries [40]. In sub-Saharan Africa, HBV (34%), HCV (17%), and alcohol (18%) are the major ones [42].

About 12.0% (range: 3.7% to 20.0%) of the CLDs in Ethiopia are because of NAFLD. This estimate is by far less than the global estimate (25.24%), while it is nearly similar to the report in the African region (13.48%) [43]. The variation in the geographical distribution of the CLD etiologies, discrepancies in the availability of diagnostic technologies, and others can rationalize the higher global NAFLD estimate as compared to our finding in Ethiopia. Wilson's disease and autoimmune hepatitis had a small contribution to CLD cases in Ethiopia, accounting for an estimated proportion of 1.0% each. However, we would like to refrain from this inference; from clinical practice experience, we doubt these etiologies are routinely investigated in Ethiopia where resources are very much limited. Unfortunately, the findings are consistent with the global report where autoimmune hepatitis and Wilson's disease accounted for 1.0% of the CLD etiologies [1, 2].

The findings in the present meta-analysis on the distribution of CLD etiologies imply the existence of the possibility of preventing, treating, and curing a large proportion of CLDs [44] in Ethiopia. The Ethiopian government has already added hepatitis B vaccines to the standard immunization schedule in the country in 2007 [45]; despite that, there are myriad barriers to ensuring wider national coverage of the vaccine in the country [46]. Thus, the authors warrant sustaining the HBV vaccination and public health promotion campaign. Since alcohol is the second major contributor of CLDs in Ethiopia, creating public awareness and government commitment in public health promotion and education programs to reduce alcohol consumption is warranted. The Ethiopian government has already made positive signs of progress too banning all advertising and lottery prizes connected with alcoholic drinks [47]. On the other hand, the magnitude of NAFLD in the Ethiopian context also needs great concern. The global trend is also depicting that the impact of viral hepatitis is expected to be overtaken by that of emergent metabolic CLDs shortly [6]. As a result, the promotion of lifestyle measures helpful in the prevention of NAFLD is recommended.

In the present review, an unidentified etiology report has contributed to a high proportion of CLD cases (45%) in Ethiopia. This might imply the need to consider other proposed risk factors, such as khat chewing [33] or availing relevant diagnostic modalities. The finding may also have been influenced by other biases, including failure of conducting a routine test for some uncommon CLD etiologies. For instance, the diagnosis of autoimmune hepatitis is based on histological abnormalities, characteristic clinical and laboratory findings (elevated serum aspartate aminotransferase [AST] and alanine aminotransferase [ALT] levels and increased serum IgG concentration), and the presence of one or more characteristic autoantibodies [48]. From clinical practice experience, the authors doubt there exists a routine test of a specific search, particularly for autoimmune hepatitis and primary biliary cholangitis in patients with CLD of unknown etiology in Ethiopia. To some extent, this might have contributed to the rate of unidentified etiology reports.

In subgroup analysis based on the study regions, the highest prevalence of HBV, HCV, and alcohol as etiology of CLD was 56.0% from the northern region, 23.0% from Addis Ababa, and 37.0% again from the northern region, respectively. A meta-analysis by Belyhun et al. also reported the highest HBV and HCV infections from the northern regions and central Ethiopia, respectively [49]. In contrast, Yazie and Tebeje reported the highest prevalence of HBV infection in Addis Ababa and lower in the northern region [50]. The discrepancies might be because of the difference in the inclusion of the participants, where only studies reporting CLD patients were included in this review. The subgroup analysis based on the study year (before vs. since 2016) showed an increase and decrease trend in the estimated pooled proportions of HBV and HCV infections in Ethiopia, respectively. This metaresult is inconsistent with the previous meta-analysis study of HBV infection in Ethiopia, where the trends of HBV infection were decreasing over the years [49]. However, the discrepancy in the findings might be attributed to the fact that the number of studies included by Belyhun et al. was larger than the studies in this review. An epidemiologic study from China has also revealed inconsistent trends where the incidence of HBV infection is decreasing while the HCV infection is increasing [51].

Worldwide, CLD is the major cause of mortality [6]. In the present review, the estimated pooled mortality rate of CLD patients in Ethiopia is 25% which is about twice as compared to the rate in the United States (12.9%) [52]. This higher mortality rate in Ethiopia could be because most CLD patients are admitted to hospitals with end-stage disease due to factors such as poverty, limited confidence in western medicine, trust in traditional medicine, or distance from hospitals [53]. Additionally, in resource-limited settings, like Ethiopia, where there is a huge shortage of specially trained clinicians and targeted treatment modalities, almost all hospitalized CLD patients get only supportive treatments [25, 34]. On the other hand, disease progression, complications, and subsequent mortality have been reported to be common in chronic HCV or HBV patients with concurrent alcohol consumption [54]. Despite this, the reported rates of these concurrent etiologies are small in the studies included in the present review [25–27, 29, 30, 34]. In this review, one of the included studies has reported hepatic encephalopathy, unidentified CLD etiology, and total bilirubin level as prognostic factors for survival [34].

### 4.1. Strengths and Limitation

To our best knowledge, this review is the first to summarize the distribution of CLD etiologies in Ethiopia and across the different regions in the country through comprehensive literature searching and analysis. It has also estimated the inpatient mortality rate in CLD patients. Such data has paramount importance for policymakers and other concerned bodies to identify the gaps for implementing and strengthening the measures required. This review is not without drawbacks; the limited number of the included studies and the use of average cut-off criteria for quality assessment scores to include primary articles in this review could be mentioned as limitations. Furthermore, all the studies included in this review are institutional-based, so the findings might not represent asymptomatic CLD patients in the community.

## 5. Conclusion

Hepatitis B virus, hepatitis C virus, and alcohol are the major identified drivers of chronic liver disease in Ethiopia and the contribution of NAFLD is also important to be worthy of attention. However, unidentified etiology report has contributed to the highest proportion of CLD cases in the country. In the present review, the estimated inpatient mortality of CLD patients in the country is also high. Based on the findings, the authors warrant the implementation and strengthening of preventive and treatment interventions for viral hepatitis B and C, further intensifying the alcohol policy of the country and promotion of interventions that prevent or reduce NAFLD cases to reduce the burden of CLDs in Ethiopia. Consideration of other proposed risk factors, such as khat chewing, and exhaustive search for the known but less frequent CLD etiologies might be helpful to lessen unknown CLD etiology reports in the country.

## Figures and Tables

**Table 1:** Critical appraisal of the primary studies using the JBI Critical appraisal checklist for an analytical cross-sectional study.

Major components	Muhie [24]	Adhanom and Desalegn [25]	Ayele and Gebre-Selassie [26]	Gaddisa Desu [27]	Taye et al. [28]	Tsega et al. [29]	Mohammed and Ali [30]	Abdelmenan et al. [31]	Orlien et al. [32]	Orlien et al.^∗^ [33]	Terefe Tesfaye [34]	Bihonegn and Ayalewu [35]
1. Were the criteria for inclusion in the sample clearly defined?	Yes	Yes	Yes	Yes	Yes	No	Yes	Yes	Yes	Yes	Yes	Yes
2. Were the study subjects and the setting described in detail?	Yes	Yes	Yes	Yes	Yes	Yes	Yes	Yes	Yes	Yes	Yes	Yes
3. Was the exposure measured validly and reliably?	Yes	Yes	Yes	Yes	Yes	Yes	Yes	Yes	Yes	Yes	Yes	Yes
4. Were objective, standard criteria used for measurement of the condition?	Yes	Yes	Yes	Yes	Yes	Yes	Yes	Yes	Yes	Yes	Yes	Yes
5. Were the confounding factors identified?	No	No	No	No	No	Yes	Yes	Yes	Yes	Yes	Yes	Yes
6. Were strategies to deal confounding factors stated?	No	Yes	No	Yes	No	Yes	Yes	Yes	Yes	Yes	Yes	Yes
7. Were the outcomes measured validly and reliably?	Yes	Yes	Yes	Yes	No	Yes	Yes	Yes	Yes	Yes	Yes	Yes
8. Was appropriate statistical analysis used?	No	No	Yes	No	No	No	No	Yes	Yes	Yes	Yes	Yes

**Table 2:** Study characteristics and populations included in the eligible studies.

Author (reference)	Study year	Study area	Study design	Study populations	Sample size	Sex		Age, yrs.
						M	F	
Muhie [24]	2019	Gondar	CS	Hospitalized adult patients with ascites.	24	NR	NR	NR
Adhanom and Desalegn [25]	2017	Addis Ababa	CS	Patients diagnosed with CLD, cirrhosis, HCC, hepatitis as labeled on the medical chart.	117	107	10	Median: 45
Ayele and Gebre-Selassie [26]	2013	Addis Ababa	CS	CLD diagnosed patients based on history, clinical, ultrasound, and impaired liver function tests.	120	76	44	Mean: 40.99 ± 14.00
Gaddisa Desu [27]	2019	Jimma	CS	Adult clinically diagnosed CLD patients admitted at medical ward and on follow-up at GI clinic with.	96	66	30	20 to 49 : 76, ≥50 : 16, 15–19 : 4
Taye et al. [28]	2014	Bale robe	CS	All patients with chronic hepatitis.	578	322	256	Mean age: 34
Tsega et al. [29]	1995	Addis Ababa	CC	The only biopsy-proven cases of patients with chronic hepatitis (14), cirrhosis (156), and HCC (68).	238	170	68	Mean: 42 ± 13.3
Mohammed and Ali [30]	2014	Addis Ababa (multicenter)	CS	Clinically diagnosed CLD patients.	117	82	55	Median = 39 (18 to 78)
Abdelmenan et al. [31]	2018	Addis Ababa	CC	CLD patients were diagnosed based on clinical features, laboratory tests, imaging techniques, and when available histological tissues assessment.	812	470	342	Mean: 40.7 ± 15.4
Orlien et al. [32]	2018	Harar	CS	Adult patients presenting for the first time with features of CLD based on the presence of suggestive clinical and ultrasound features.	150	108	42	Median: 30
Orlien et al.^∗^ [33]	2018	Harar	CC	Adult patients presenting for the first time with features of CLD based on the presence of suggestive clinical and ultrasound features.	150	108	42	Median (IQR): 30 (25–40)
Terefe Tesfaye et al. [34]	2019	Multicenter^a^	CS	Hospitalized adult CLD patients diagnosed based on suggestive clinical and/or ultrasound findings.	119	85	24	Median (IQR): 38(30–40)
Bihonegn and Ayalewu [35]	2019	Mekelle	CC	CLD (cases) and non-CLD (controls) patients who were attending in the GI unit.	94	77	17	Mean: 41 ± 13

^a^Multicenter: Jimma, Addis Ababa, and Harar, CC: case-control, CS: cross-sectional, IQR: interquartile range, CLD: chronic liver disease, HCC: hepatocellular carcinoma.

**Table 3:** The overall summary of reported frequencies of CLD etiologies in the primary studies included in this review, Ethiopia, 2021.

First author [reference]	Sample size	Hepatitis B virus	Hepatitis C virus	Hepatosplenic schistosomiasis	Co-hepatitis B and C virus	Co-hepatitis B and D virus	Visceral leishmaniasis	HIV	Alcohol	NAFLD	Wilson's disease	Autoimmune hepatitis	Biliary cirrhosis	Alcohol plus HBV	Alcohol plus HCV	Unidentified
Muhie [24]	24	8	3	NR	NR	NR	NR	NR	4	0.0	1	NR	NR	NR	NR	NR
Adhanom and Desalegn [25]	117	55	21	NR	3	NR	NR	NR	2	NR	NR	NR	NR	NR	NR	51
Ayele and Gebre-Selassie [26]	120	43	27	NR	3	NR	NR	NR	NR	NR	NR	NR	NR	NR	NR	NR
Gaddisa Desu [27]	96	30	7	NR	1	NR	NR	NR	20	NR	NR	NR	NR	2	1	44
Taye et al. [28]	578	80^a^	8^b^	NR	NR	NR	NR	NR	NR	NR	NR	NR	NR	NR	NR	NR
Tsega et al. [29]	238	65	91	NR	1	15^c^	NR	NR	NR	NR	NR	NR	NR	NR	NR	NR
Mohammed and Ali [30]	117	40	22	NR	3	NR	NR	11	NR	NR	NR	NR	NR	NR	NR	NR
Abdelmenan et al. [31]	812	467	138	39	NR	NR	NR	NR	117	163	NR	NR	NR	NR	NR	NR
Orlien et al. [32]	150	55	2	4	NR	NR	1	NR	3	NR	NR	2	NR	NR	NR	83
Orlien et al. ∗ [33]	150	55	2	4	NR	NR	1	3	3	NR	NR	2	NR	NR	NR	80
Terefe Tesfaye et al. [34]	109	39	12	7	1	NR	NR	NR	15	4	1	1	1	NR	NR	28
Bihonegn and Ayalewu [35]	94	73	15	NR	NR	NR	NR	NR	53	NR	NR	NR	NR	NR	NR	NR

Assessed from ^a^ 358 participants, ^b^ 220 participants, ^c^ 65 participants. HIV: human immunodeficiency virus, NR: not reported, CLD: chronic liver disease, HBV: hepatitis B virus, HCV: hepatitis C virus NAFLD: nonalcoholic fatty liver disease.

**Table 4:** Estimated proportion of chronic liver disease etiologies and heterogeneity of the estimates in Ethiopia, 2021.

Etiologies of CLD	No. of studies	Sample size	Frequency	Pooled estimate [95% CI]	Q	*I* ^2^	*p*-value
*Infectious etiology*							
Hepatitis B virus	11	2245	955	40.0 [29.0,51.0]	270.9	96.3	<0.001
Hepatitis C virus	11	2107	346	15.0 [9.0, 21.0]	240.4	95.8	<0.001
Schistosomiasis	3	127	50	4.0 [1.0, 6.0]	2.02	1.1	0.36
HIV	2	267	14	5.0 [−2.0, 13]	6.38	84.3	0.01
Co-HBV and HCV	4	570	8	1.0 [0.0, 2.0]	3.7	18.9	0.30

*Noninfectious etiology*							
Alcohol	7	1412	214	17.0 [9.0, 25.0]	184.6	96.7	<0.001
NAFLD	2	931	167	12.0 [4.0, 28.0]	51.6	98.0	<0.001
Wilson's disease	2	143	2	1.0 [−1.0, 3.0]	0.6	0.00	0.44
AIH	2	269	3	1.0 [−0.0, 2.0]	0.1	0.00	0.75
Unidentified	5	506	214	45.0 [34.0, 56.0]	32.08	87.53	<0.001

CLD: chronic liver disease, HBV: hepatitis B virus, HDV: hepatitis D virus, NAFLD: nonalcoholic fatty liver disease, AIH: autoimmune hepatitis, I^2^: heterogeneity, *Q*: Cochran's Q.

**Table 5:** Bivariate and multivariate meta-regression analysis of factors associated with the heterogeneity of the three most commonly reported chronic liver disease etiology estimates among chronic liver disease patients in Ethiopia, 2021.

Variables	*β*-coefficient	95% CI	*p* value
*Bivariate regression*			
Hepatitis B virus			
Age	0.0086	−0.0207, 0.0792	0.56
Sample size	0.0001	−0.0003, 0.0006	0.60
Hepatitis C virus			
Age	0.0166	0.0049, 0.0283	0.01
Sample size	0.0001	−0.0002, 0.0003	0.68

*Multivariate regression for hepatitis C virus estimate*			
Age	0.0165	0.0016, 0.0314	0.03
Sample size	8.66e-^08^	−0.0002, 0.0003	0.99

**Table 6:** Summary of subgroup analysis of the three most commonly reported chronic liver disease etiology estimates in terms of study regions and publication/study year in Ethiopia, 2021.

Variables	No. of studies	Estimates	95% CI	*p* value
*Hepatitis B virus*				
Addis Ababa	5	40.0	26.0, 55.0	0.32
Northern region	2	56.0	13.0, 100.0	
Oromia	4	31.0	23.0, 39.0	
Before 2016	4	29.0	23.0, 35.0	0.01
Since 2016	7	46.0	34.0, 58.0	

*Hepatitis C virus*				
Addis Ababa	5	23.0	15.0, 30	<0.001
Northern region	2	15.0	9.0, 22.0	
Oromia	4	5.0	1.0, 8.0	
Before 2016	4	21.0	4.0, 37.0	0.33
Since 2016	7	12.0	5.0, 19.0	

*Alcohol*				
Addis Ababa	2	8.0	−4.0, 21.0	0.38
Northern region	2	37.0	−2.0, 76.0	
Oromia	3	12.0	−0.0, 24.0	

## Data Availability

The data used to support the findings of this study are included within the article.

## Abbreviations

AIH: Autoimmune hepatitis
CC: Case-control
CLD: Chronic liver disease
CS: Cross-sectional
HBV: Hepatitis B virus
HCC: Hepatocellular carcinoma
HDV: Hepatitis D virus
HIV: Human immunodeficiency virus
Q: Cochran's Q
*I* ^2^: Heterogeneity
NAFLD: Nonalcoholic fatty liver disease
NR: Not reported
PRISMA: Preferred reporting items for systematic reviews and meta-analyses.
